# Not transplanting kidney donors with acute kidney injury: a missed opportunity?

**DOI:** 10.1590/2175-8239-JBN-2019-0194

**Published:** 2019-12-20

**Authors:** Naoka Murakami, Leonardo V. Riella

**Affiliations:** 1 Harvard Medical School, Brigham & Women’s Hospital, Transplantation Research Center, Boston, MA, United States of America.

Kidney transplantation is the treatment of choice for end-stage renal disease (ESRD). In the US, studies have shown a clear survival benefit of transplantation even when the recipients are HLA-incompatible with positive cross-matches, compared with remaining on the waitlist^1^. However, organ shortage has been a universal challenge for the transplant field and the number of patients on the waitlist continues to rise. In order to expand the organ donor pool, several approaches have been taken, including kidney swap programs for sensitized patients or ABO-incompatible living donor-recipient pairs. For deceased donors, the use of expanded criteria donors, transplantation from HCV-positive donors to HCV-negative recipients, and use of HIV-positive organs have been implemented. Despite these efforts, the organ discard rate is still high at about 20% of all organ offers^2^, and the rate is higher in donors with acute kidney injury (AKI). The most common reason for turning down organ offers is the “inadequate” quality of the organs based on biopsy, followed by either AKI or expanded criteria donor with significant kidney risk factors. This tendency continues after incorporation of a new kidney allocation policy in the US in 2014. Therefore, it is of particular importance to determine long-term outcomes of recipients who received kidneys from AKI donors and to establish the risk factors for post-transplant kidney dysfunction. 

In this issue of the BJN, Takase^3^ and colleagues retrospectively reviewed their single-center experience of kidney transplantation from deceased donors with severe AKI. Among 732 consecutive deceased donor kidney transplant cases between 2010 and 2018, four cases with pre-procurement creatinine levels above 6.0 mg/dL were identified. In all four cases, donors were young (range 35-43), initial creatinine at presentation was normal, all had acute rhabdomyolysis as a cause of AKI, and pre-donation biopsy showed acute tubular necrosis (ATN) without glomerular, tubular, or vascular abnormalities. Cold ischemia time was long in all cases, ranging from 20 to 25 hours. Though all cases were complicated by delayed graft function (DGF), all organs were successfully transplanted and recipients’ allograft function was excellent [range 48-98 mL/min/1.73m^2^] at the latest follow-up (range 5 months to 2.7 years post-transplant). 

The utility of kidneys from deceased donors with AKI has gained more attention recently, in accordance with the interest to expand the donor pool and to lower the organ discard rate. It has been reported that the discard rate increases stepwise with higher AKI stages, with Acute Kidney Injury Network (AKIN) stage 3 having an odds ratio for discard of 2.7^4^. Recent study by Heilman et al. analyzed 1,313 transplanted kidneys, of which 934 (75.7%) had AKI and 447 (34%) had AKIN stage 3 AKI. They showed that stages of AKI did not affect long-term kidney survival (median follow-up 3-4.5 years post-transplant), though the incidence of DGF was significantly higher in recipients who received kidneys from AKIN stage 2 and 3 donors^5^. Similarly, Hall et al. conducted a multi-center retrospective study of 2,430 kidneys (585 kidneys (24%) with AKI, 85 (3.5%) with AKIN stage 3 AKI) and suggested that there was no difference in composite 3-year graft survival associated with AKI stages^6^. On the other hand, a study using transplant registry in UK (n=11,646; 1,869 (6.0%) with AKI and 172 (1.5%) with AKIN stage 3 AKI) reported slightly lower graft survival 1-, 3-, and 5-year post-transplant in AKI group compared with non-AKI group^7^, which the authors discussed might not be clinically relevant (for example, 5-yr graft survival of 78% in non-AKI vs. 76% in AKI group) (Table 1). Nevertheless, these studies were observational and we should be cautious on extrapolating the results to all AKI donors, since the analyses only included the organs that physicians considered “viable” for transplantation despite AKI, and could potentially overestimate the outcomes. 

**Table 1:** Graft failure and DGF incidence based on donor AKIN stages

		Analysis follow-up length		AKIN 0/No AKI	AKIN 1 Cr increase >0.3 mg/dL or to 1.5-1.9 times baseline	AKIN 2 2-2.9 times baseline	AKIN 3 3.0 times baseline. Cr >4.0 mg/dL or initiation on RRT
Heilman et al.^4^	U.S. (Single-center)	Median 4 years	Graft Failure (HR)	1.0 (ref)	0.97 (0.67-1.40)	0.74 (0.45-1.24)	0.70 (0.45-1.10)
			DGF Incidence (%)	33.90%	33.5%	44%	75.4%
Hall et al.^5^	U.S (Multi-center)	3 years	Graft Failure (HR)	1.0 (ref)	0.92 (0.73-1.14)	0.73 (0.48-1.11)	0.92 (0.58-1.47)
			DGF Incidence (%)	39%	56%*		
Boffa et al.^6^	U.K. (UK Transplant Registry)	1 year	Graft Failure (HR)	1.0 (ref)	1.20 (1.03-1.41)*		
			DGF Incidence (%)	28% (1.00)	35% (1.15-1.54)	43% (1.37-2.03)	55% (2.25-3.96)

* All AKI stages combined. HR: hazard ratio (95% Confidence Interval). AKI: acute kidney injury, Cr: creatinine, DGF: delayed graft function AKIN stages based on serum creatinine are shown (refer to AKI Network website (www.AKINET.org) for full definition).

How can we best determine whether the organs from AKI donors should be considered for transplantation or not? As Heilman et al. suggested, several studies explored the use of pre-procurement biopsy for guidance; two biopsy scoring systems have been proposed to evaluate the quality of kidney before procurement: Banff scoring system and Remuzzi score. Data suggest using the value of pre-procurement pathology findings to predict outcomes. However, decision-making based on histological findings comes with challenges such as significant inter-observer variability in pathology readings, different pathology preparation (frozen section vs. paraffin section) and necessity of urgent reading, which might result in a below-optimal histological interpretation. 

At the same time, we should keep in mind that local logistical factors and allocation policy play a great role in organ distribution. In Brazil, there is a documented higher incidence of AKI in ICU and limited resources in organ transportation compared to other countries. For example, once the patients are classified as brain-dead, renal perfusion support is not commonly provided, which could precipitate kidney injury. In addition, after procurement, the organ is often transported for long distances, across states^8^, using local transportation lines and not on perfusion pump, which prolongs cold ischemia time and inevitably increases the risk of further kidney injury. This is a relatively common situation in developing countries; strategies to best allocate and maximize organ offers in places with limited resources are needed.

In conclusion, it is noteworthy that AKI organs may have acceptable graft survival in the long term. It is crucial to individualize the risk evaluation when using these organs based on the cause of AKI, ischemic time, size/age mismatch, and immunological risks. Lastly, pre-transplant donor biopsy may also serve as a complementary tool for the final decision by excluding scarring and other irreversible injuries.
